# Transcriptomic dynamics of ABA response in *Brassica napus* guard cells

**DOI:** 10.1007/s44154-024-00169-7

**Published:** 2024-10-14

**Authors:** Florent Villiers, Yasir Suhail, Jade Lee, Felix Hauser, Jaeung Hwang, Joel S. Bader, John K. McKay, Scott C. Peck, Julian I. Schroeder, June M. Kwak

**Affiliations:** 1 Centre de Recherche de La Dargoire, Bayer CropScience, 69009 Lyon, France; 2 Department of Biomedical Engineering, Johns Hopkins University, Baltimore, MD 21205 USA; 3 Department of Cell Biology and Molecular Genetics, University of Maryland, College Park, MD 20742 USA; 4 Division of Biological Sciences, Cell and Developmental Biology Department, University of California San Diego, La Jolla, CA 92093 USA; 5 Department of New Biology, DGIST, Daegu, 42988 Republic of Korea; 6 Department of Bioagricultural Sciences, Colorado State University, Fort Collins, CO 80523-1177 USA; 7 Department of Biochemistry and Christopher S. Bond Life Sciences Center, Interdisciplinary Plant Group, University of Missouri, Columbia, MO 65211 USA

**Keywords:** Abscisic acid, Guard cells, Rapeseed, Stomatal movement, Transcriptome

## Abstract

Drought has a significant, negative impact on crop production; and these effects are poised to increase with climate change. Plants acclimate to drought and water stress through diverse physiological responses, primarily mediated by the hormone abscisic acid (ABA). Because plants lose the majority of their water through stomatal pores on aerial surfaces of plants, stomatal closure is one of the rapid responses mediated by ABA to reduce transpirational water loss. The dynamic changes in the transcriptome of stomatal guard cells in response to ABA have been investigated in the model plant *Arabidopsis thaliana*. However, guard cell transcriptomes have not been analyzed in agronomically valuable crops such as a major oilseed crop, rapeseed. In this study, we investigated the dynamics of ABA-regulated transcriptomes in stomatal guard cells of *Brassica napus* and conducted comparison analysis with the transcriptomes of *A. thaliana*. We discovered changes in gene expression indicating alterations in a host of physiological processes, including stomatal movement, metabolic reprogramming, and light responses. Our results suggest the existence of both immediate and delayed responses to ABA in Brassica guard cells. Furthermore, the transcription factors and regulatory networks mediating these responses are compared to those identified in Arabidopsis. Our results imply the continuing evolution of ABA responses in Brassica since its divergence from a common ancestor, involving both protein-coding and non-coding nucleotide sequences. Together, our results will provide a basis for developing strategies for molecular manipulation of drought tolerance in crop plants.

**Supplementary Information:**

The online version contains supplementary material available at 10.1007/s44154-024-00169-7.

## Introduction

Desiccation of crops during drought causes severe damage and lost yields. The annual loss caused by drought alone range between 30 and 90%, depending on crop species, more than the sum of all pathogen losses (Boyer 1982). Drought damage costs the US an average $9.4 billion annually (Federal Emergency Management Agency, 1995; García-León et al. 2021). Climate change is predicted to intensify extreme climate events, including drought (Dai 2012; Trenberth et al. 2013), and could make crop production more vulnerable to even moderate droughts. Moreover, freshwater scarcity is predicted to be a major problem for this century. Globally, 65% of fresh water is used for agriculture and plants. By 2050, water demand for agriculture could double, while the availability of fresh water is predicted to drop by 50%, owing to climate change. (Gupta et al. 2020; Mekonnen & Hoekstra 2014). Therefore, it is imperative to understand and develop strategies to improve water use efficiency during crop production.

Plants respond to drought and water deficit by evoking necessary cellular events mediated by the plant hormone abscisic acid (ABA). A wave of sequential molecular responses throughout the entire plant results in adaptation to the reduction in water availability for plant survival (Gupta et al. 2020). Although plants employ a variety of different mechanisms under these conditions, one of the first processes is the prevention of excessive water loss. Typically, plants lose approximately 95% of their water via transpiration through stomatal pores in the leaf epidermis. Stomatal pores are formed by a pair of guard cells that regulate the pore size, thereby controlling water loss and CO_2_ uptake under diverse environmental conditions. Water deficit response mediated by ABA results in reduced water loss by decreasing the stomatal aperture. Reduced stomatal conductance sustains plant survival, but comes at the cost of reduced photosynthetic assimilation and plant growth by impeding the uptake of CO_2_ (Gupta et al. 2020; Rodrigues et al. 2019). These responses in leaves have been shown to involve both ABA-dependent and ABA-independent pathways (Shinozaki & Yamaguchi-Shinozaki 1996). Moreover, ABA signaling mediates osmotic stress response (Lozano-Juste et al. 2020; Yoshida et al. 2014). In the ABA-dependent pathway of osmotic stress response, ABA-activated SnRK2 kinases mediate numerous subsequent cellular events. Recent studies have shown that RAF-like MAPKKKs activate SnRK2s in osmotic stress signaling (Fabregas et al. 2020). In addition, some genes are activated by both the ABA-dependent and ABA-independent pathways, with considerable cross-talk (Nakashima et al. 2014; Narusaka et al. 2003).

Guard cells have become a single cell model for understanding rapid environmentally-induced signal transduction in plants. The study of guard-cell-specific signal transduction has been a cornerstone in the discovery of molecular components underlying stomatal movements, and thus in drought resistance, that cannot be addressed by employing whole-organism approaches. To fully understand the dynamic cellular networks that respond to drought, it is necessary to obtain detailed information on changes in transcriptomes in a time-dependent manner and to use this information to enable network analyses at the systems level. Responses monitored in heterogeneous tissues may reflect mixtures of unique responses in individual cell types. Therefore, to fully understand signaling pathways, profiling of cell type-specific samples is required, which can later be placed in a more integrated context including neighboring cells and additional contributing factors of the signaling process such as hydraulic changes.

*Brassica napus* is an important food crop as a source of edible oil, popularly known as canola or rapeseed oil. *B. napus* has been bred to lower the total content of the anti-nutrients such as erucic acids, eicosenoic acids, and glucosinolates, and serves as a major food source (Jonnson 2009; Kondra & Stefansson 1965; Qiu et al. 2006). *B. napus* originated from the polyploid hybrid speciation of *Brassica rapa* and *Brassica oleracea* (Allender & King 2010; Song & Osborn 1992). The synonymous nucleic acid substitution rates (Chalhoub et al. 2014) and low degree of chromosomal rearrangements indicate a relatively recent speciation event about ten thousand years (Parkin et al. 1995). Of the 19 chromosomes in *B. napus*, 10 chromosomes are derived from (subgenome A) of *B. rapa* and 9 chromosomes (subgenome C) from *B. oleracea*. The *Brassica* species, along with the model plant *A. thaliana*, are members of the Brassicaceae family. It is estimated that the split between Arabidopsis and Brassica occurred 10–20 million years ago, and a number of genome duplication events have since occurred, giving rise to the Brassica species from its common ancestor with Arabidopsis (Blanc et al. 2003; Ermolaeva et al. 2003; Yang et al. 1999).

The numerous studies of drought stress (Kuromori et al. 2022; Zhu 2002) and ABA responses (Chen et al. 2020; Hsu et al. 2021; Komatsu et al. 2020) in *A. thaliana* and its genetic similarity with *B. napus* allow us to use this existing knowledge to compare and interpret our results from *B. napus*. The present study evaluates the genome-wide transcriptomic response to ABA in the guard cells of *B. napus*. The plasticity of drought avoidance traits in the leaf, specifically the control of stomatal conductance, was analyzed in this study. We compare the regulation of gene expression in *B. napus* with the known biology of *A. thaliana*, and draw conclusions regarding the evolution of the ABA response.

## Results and Discussion

### Dynamic transcriptome analysis of *Brassica napus* stomatal guard cells upon ABA treatment

Guard cells isolated from 7-week-old *B. napus* (DH12075) leaves with purity of over 99% were subjected to 15-min, 60-min, and mock ABA treatment respectively (Fig. S1). In order to achieve sufficient yields of RNA from highly-purified protoplast samples, three to five protoplast isolations were combined to form one biological sample. Three biological samples, each including the three different treatment conditions, were prepared and used for transcriptomics analysis.

All RNA-seq reads (100 bp single ended) were aligned to the *B. napus* genome of the Darmor line (Chalhoub et al. 2014) with TopHat2 (Kim et al. 2013). Only reads unambiguously mapping to single genes were counted with HTSeq (Anders et al. 2015) to distinguish between the levels of expression of paralogs, and correctly call differentially expressed genes.

We were able to map approximately 100 million reads for each sample, to a total of 78,105 genes out of 101,040 annotated in the *B. napus* genome. It is possible that the other genes are not expressed in the guard cells of our line, or that no read is specifically mapped to them due to polyploidy. In addition, no alternatively spliced transcripts were included in the gene models. The statistical error in the read counts is modeled using the negative binomial probability distribution. Fig. S2 confirms that the negative binomial distribution (whose significance is discussed in the methods) is a better fit for the noise in the read counts than the Poisson distribution. The baseline gene expression in our dataset is roughly similar to that expected from single-cell RNA-seq analysis of stomatal guard cells (Fig. S3).

## Differential expression analysis

We estimated the differential expression of all genes in *B. napus* guard cells at 15 min and 60 min of ABA treatment versus the mock-treated control. Both time points combined yield a total of 12,228 non-redundant genes (12%) responsive to ABA with a false discovery rate of less than 0.05. By measuring gene expression at two different times, we determined which genes showed grouped patterns of temporal response to ABA: short-lived (only at 15 min), delayed (only at 60 min), increasing with time, or unchanged.

**Fig. 1:**
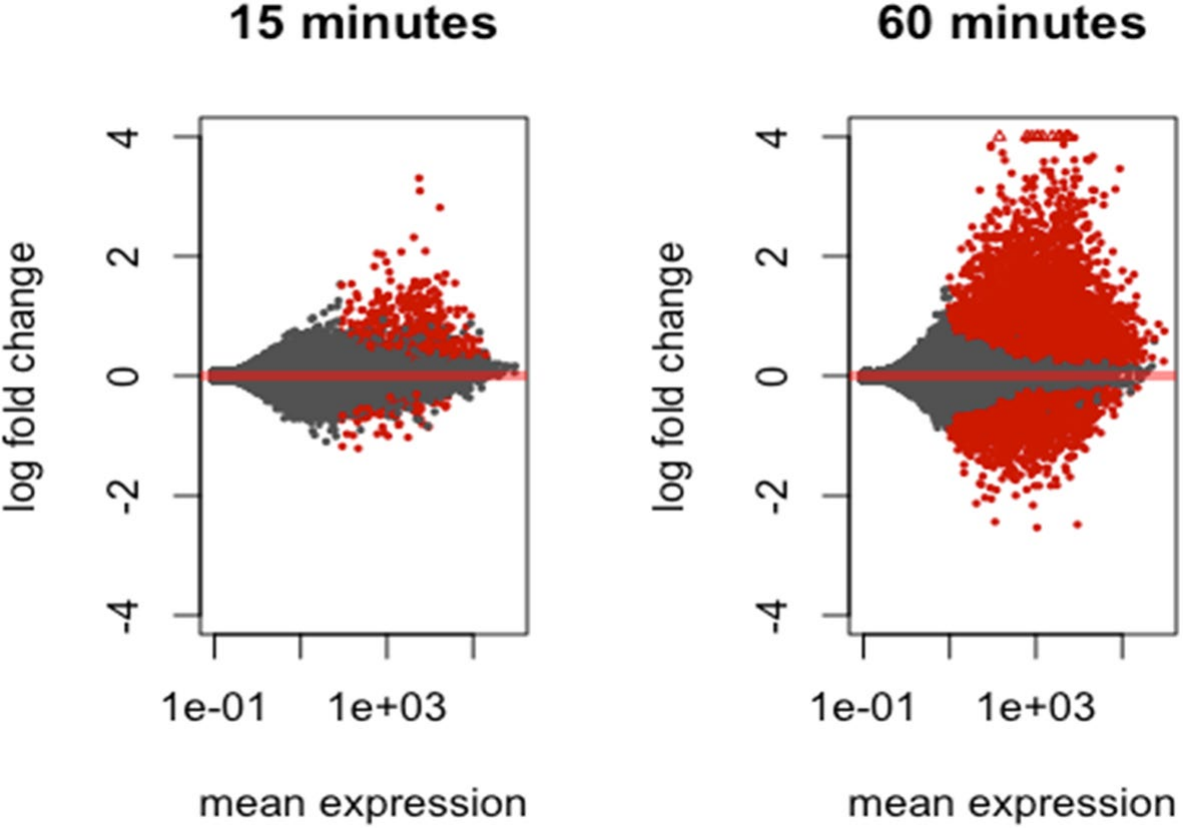
MA plot showing the log ratio (M) versus the average read count (A) for 15 min and 60 min. The red colored dots denote genes identified as significantly differentially expressed. The black colored dots represent genes that are not identified as significantly differentially expressed

The behavior of the detected transcriptome at these time points is visualized in Fig. 1, which shows the distribution of log2 fold changes at 15 min and 60 min. This visualization, also known as the Bland–Altman plot (Altman & Bland 1983; Bland & Altman 1999), confirms that the mean log fold change is zero for genes with both low and high basal gene expression and that no further normalization is required. As expected, the number of genes for which expression is significantly affected by ABA is higher at 60 min than at 15 min (12,216 vs. 479 respectively; Fig. 1, Table 1). On average, larger fold-changes are also observed at 60 min (Fig. 1).

**Table 1:** Contingency table showing the number of genes with significant positive and negative differential expression (< 5% FDR) for 15 min and 60 min of ABA treatment

	Down-regulated at 60 min	Non-significantly regulated at 60 min	Up-regulated at 60 min
Down regulated at 15 min	64	7	0
Non-significantly regulated at 15 min	4282	89,104	7177
Up regulated at 15 min	0	4	402

The relationship between expression at 15 min and 60 min for individual genes is visualized in Fig. S4. The linear regression shows a ${R}^{2}$ of $0.2736097$ and a highly significant *p*-value < 2.2 × 10^–16^ when comparing the two treatment times, demonstrating that gene expression levels at the two time points are correlated. Therefore, genes with more extreme log2-fold changes show more correlated differential expression between the response at 15 min and 60 min. This result is expected because transcripts that are not regulated are not anticipated to show a correlation between their expression levels for different times of ABA exposure.

Since we are primarily interested in genes that are significantly differentially expressed, the number of genes against each direction of regulation is tabulated at each time point in Table 1. Nearly all genes identified as differentially expressed at t = 15 min are also differentially expressed at t = 60 min in the same direction. The log2-fold changes are generally higher at 60 min, giving the test at t = 60 min higher statistical power. For many genes, the effect size at 60 min makes it easier to detect their differential expression (i.e. at 15 min the effect size may not be much above background noise).

## Temporal dynamics suggest role for genes in the early response to abscisic acid

**Fig. 2:**
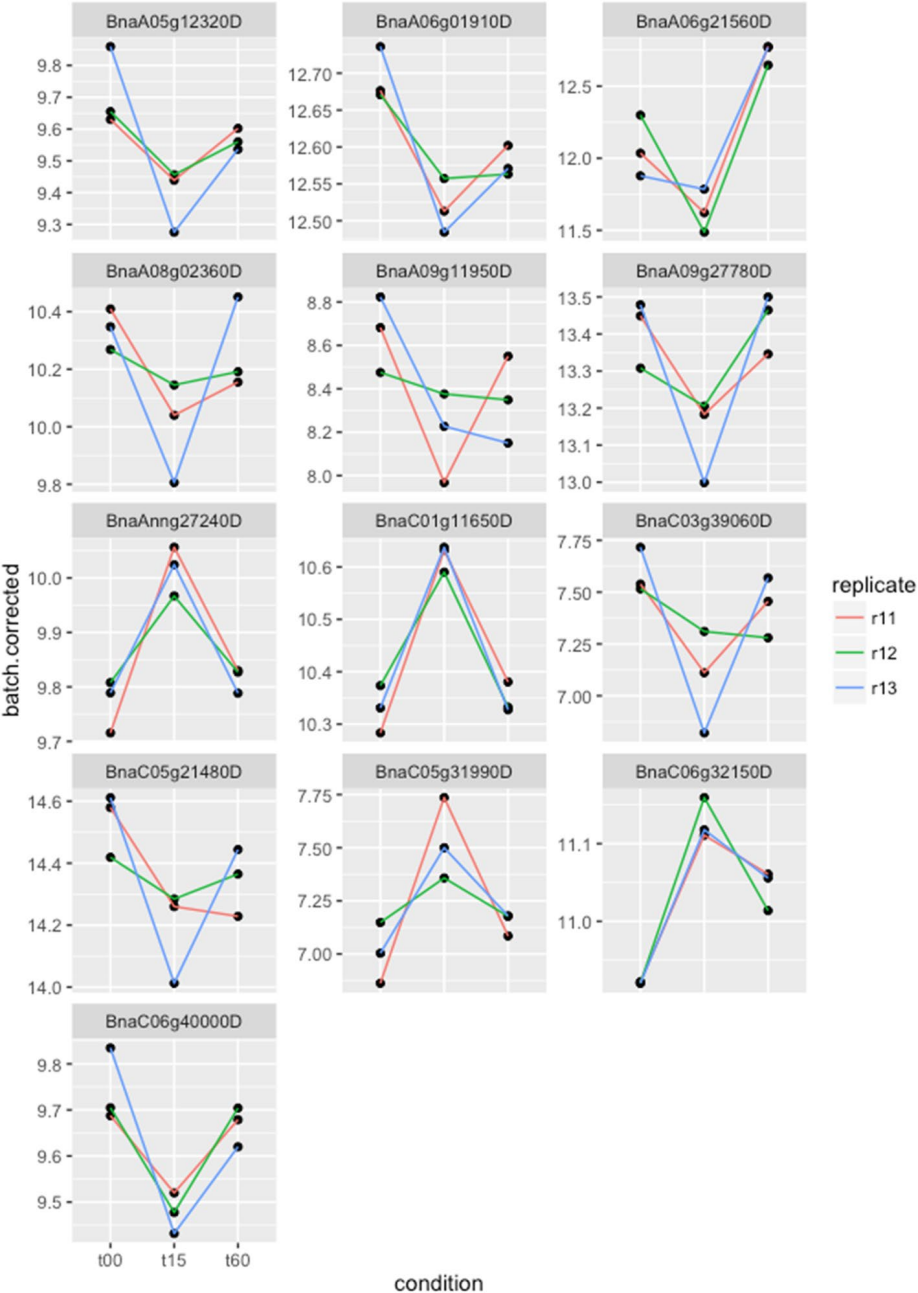
Batch corrected read counts for genes showing transient regulation at 15 min compared to 60 min, with a greater activation/repression at the early time point. The predicted annotations of the *Brassica* genes and their corresponding Arabidopsis genes are listed in Table 2

Comparing temporal dynamics of transcripts, 13 genes were found to be regulated to a lesser extent at 60 min than at 15 min, indicating early and transient up- or down-regulation upon ABA stimulation. The levels of these genes for the 3 time points (after batch correction for easier visualization) are plotted in Fig. 2.

Table 2 lists the genes that are rapidly regulated at 15 min of exposure to ABA before returning to steady-state levels by 60 min. Although only a few genes exist for this category, they hint at an initial transient response to ABA. Examination of the literature provides indications for possible roles for some of these genes. *FAB1C*, a predicted 1-phosphatidylinositol-3-phosphate 5-kinase, is known to be responsible for fast closure of the stomata, and a mutation in this gene causes slow stomatal closure (Bak et al. 2013). This role would be consistent with our observation that *FAB1C* is up-regulated during this initial response and subsequently returns to basal levels without a role in the later ABA response. In context of the role of ABA in seed maturation and dormancy, *CYP707A1* is known to be expressed in mid-maturation and is then down-regulated in late maturation (Okamoto et al. 2006). *CYP707A1* encodes an abscisic acid 8’-hydroxylase, a key enzyme in the oxidative catabolism of ABA (Kushiro et al. 2004). Perhaps a similar feedback mechanism could be activated upon treatment of guard cells with ABA, which may explain the initial up-regulation and then return to basal expression of *CYP707A1*.

**Table 2:** Genes exhibiting transient regulation within the first hour of ABA treatment

Temporal Pattern	*B. napus* gene	*A. thaliana* gene	Common name	FDR at 15 min	FDR at 60 min
Only down-regulated at 15 min	BnaA05g12320D	AT2G30040	MAPKKK14	0.00986	0.195
Only down-regulated at 15 min	BnaA08g02360D	AT1G49850		0.03198	0.683
Only down-regulated at 15 min	BnaA09g11950D	AT1G64090	RTNLB3	0.02868	0.071
Only down-regulated at 15 min	BnaA09g27780D	AT1G27730	STZ	0.00735	0.814
Only down-regulated at 15 min	BnaC03g39060D	AT3G15353	ATMT3	0.02381	0.718
Only down-regulated at 15 min	BnaC05g21480D	AT1G27730	STZ	0.00029	0.889
Only down-regulated at 15 min	BnaC06g40000D	AT1G79660		0.02328	0.485
Only up-regulated at 15 min	BnaAnng27240D	AT4G19230	CYP707A1	0.00192	0.074
Only up-regulated at 15 min	BnaC01g11650D	AT4G19230	CYP707A1	0.01032	0.536
Only up-regulated at 15 min	BnaC05g31990D			0.01387	0.088
Only up-regulated at 15 min	BnaC06g32150D	AT1G71010	FAB1C	0.01980	0.513

## Gene regulation in gene families is largely conserved from the ancestor

Much is known about ABA signaling in Arabidopsis guard cells. To make use of the existing literature on *A. thaliana* genes, we mapped *B. napus* genes to their corresponding closest *A. thaliana* (syntenic or non-syntenic) homologs. *B. napus* is the product of a number of genome duplication and fractionation events since diverging from its most recent common ancestor with *A. thaliana*. Therefore, multiple *B. napus* genes map to the same *A. thaliana* ortholog. For the purposes of this study, these groups of orthologous *B. napus* genes are considered to be gene families resulting from the duplication of a shared ancestral gene.

It is generally thought that gene duplication through evolution allows for specialization of the supernumerary copies, either spatially, temporally or functionally. To investigate the evolution of drought responses in *B. napus*, we assessed a possible divergence in the response of individual *B. napus* genes compared to their Arabidopsis counterparts. Most gene families do not appear to have diverged, possibly due to insufficient evolutionary time: Fig. S5A shows a consistent overall regulation of *B. napus* gene family members with each other, as well as with their Arabidopsis ortholog(s). Groups of paralogous genes (assumed to be evolved from the same ancestral gene) are observed to have correlated genetic expression, suggesting conservation of the regulatory response. A few gene families have a higher standard deviation of their gene regulation (Fig. S5B), eight of which show a particularly high inconsistency within their members (SD > 1.6, Table 3).

**Table 3:** Table of B. napus genes with the most divergent fold changes in their gene families. All gene families with a standard deviation of their fold changes at 60 min of ABA treatment more than $1.6$ were selected and their member genes listed

#	A. thaliana Ortholog	*B. napus* gene	*A. thaliana* common name	log2 (Fold Change)	Adjusted *p*.-value
1	AT4G21060	BnaA01g10960D	AtGALT2	-0.2087579	0.17604
	AT4G21060	BnaC01g12450D	AtGALT2	-0.2131616	0.32058
	AT4G21060	BnaC07g36550D	AtGALT2	3.3005180	< 2e-16
	AT4G27410	BnaA01g16400D	ANAC072	3.6033603	< 2e-16
2	AT4G27410	BnaA03g48570D	ANAC072	3.3041495	< 2e-16
	AT4G27410	BnaC01g19550D	ANAC072	0.1300110	0.70897
	AT4G27410	BnaC07g40860D	ANAC072	3.1791690	< 2e-16
	AT3G15670	BnaA01g28600D	NA	0.6381671	NA
3	AT3G15670	BnaA05g23860D	NA	4.3448604	< 2.22e-16
	AT3G15670	BnaC01g35900D	NA	0.0754009	NA
	AT3G15670	BnaC03g39230D	NA	0.0512065	NA
	AT3G15670	BnaC05g37670D	NA	4.0675792	< 2.22e-16
	AT3G02480	BnaA01g32930D	NA	-0.0509738	NA
4	AT3G02480	BnaA03g27910D	NA	4.4150238	< 2.22e-16
	AT3G02480	BnaC03g32950D	NA	2.2319576	4.1865e-12
	AT3G17520	BnaA03g34560D	NA	0.4042299	NA
5	AT3G17520	BnaAnng35040D	NA	5.0627122	< 2.22e-16
	AT3G17520	BnaC03g40050D	NA	1.4258471	2.1121e-05
	AT3G17520	BnaC05g35990D	NA	3.5844242	< 2.22e-16
	AT4G34020	BnaA03g50810D	AtDJ1C	3.8242444	< 2e-16
6	AT4G34020	BnaAnng26560D	AtDJ1C	0.2359967	0.478637
	AT4G34020	BnaC01g04310D	AtDJ1C	0.3422487	0.090944
	AT4G34020	BnaC01g04320D	AtDJ1C	-0.0557400	NA
7	AT2G47770	BnaA04g29550D	ATTSPO	2.7599247	< 2.22e-16
	AT2G47770	BnaA05g00220D	ATTSPO	1.9034236	7.381e-16
	AT2G47770	BnaC04g00110D	ATTSPO	0.1170079	0.58354
	AT2G47770	BnaC04g51120D	ATTSPO	4.2157486	< 2.22e-16
8	AT1G69260	BnaA07g24330D	AFP1	3.7297320	< 2.22e-16
	AT1G69260	BnaA07g27800D	AFP1	0.6839890	NA
	AT1G69260	BnaC06g25430D	AFP1	4.1553036	< 2.22e-16
	AT1G69260	BnaC06g30430D	AFP1	0.0644924	NA

Group 6, for instance, contains four members, three of which do not significantly react to ABA stimulation while a fourth member (BnaA03g50810D) is more than 14-fold up-regulated. Interestingly, datasets from AtGeneExpress also identify the corresponding Arabidopsis ortholog (AT4G34020) as ABA-activated, revealing a conserved function for this duplicated gene (Goda et al. 2008). Conversely, group 2 has three members that are highly regulated by ABA (9 to 12-fold up-regulation), with a fourth member not statistically different during ABA-treatment compare to mock-treated GC. The corresponding Arabidopsis gene (AT4G27410, RESPONSIVE TO DESICCATION 26) encodes a NAC transcription factor induced in response to desiccation that acts as a transcriptional activator in ABA-mediated dehydration response (Fujita et al. 2004).

## Many known ABA signaling genes are up-regulated at both 60 min and 15 min

**Fig. 3:**
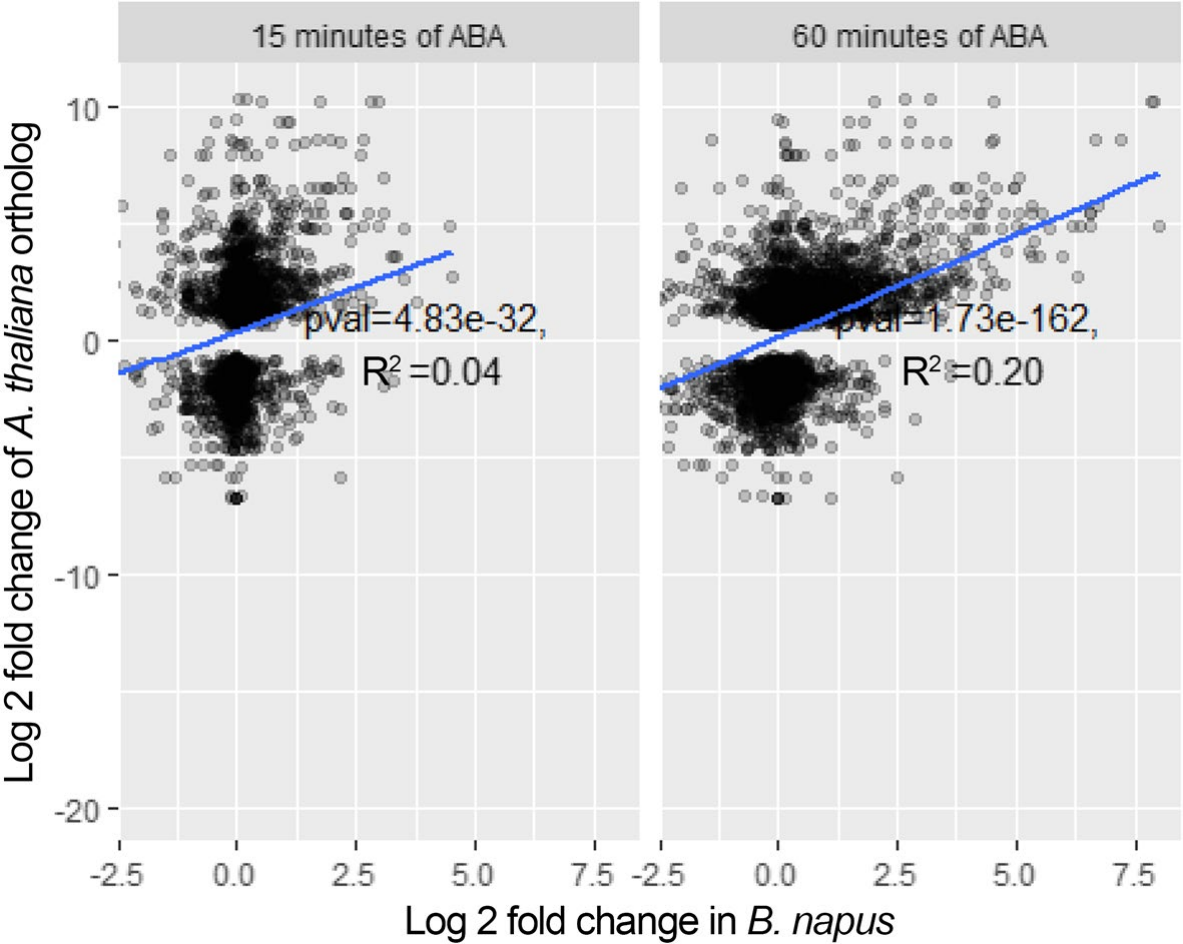
Statistically significant correlation of the ABA response in *A. thaliana* and *B. napus* guard cell protoplasts. The log2 fold change observed in *B. napus* after 15 min and 60 min of ABA treatment is plotted against that of the corresponding Arabidopsis ortholog after 3 h of ABA treatment as reported in Wang et al. (2011). Only significantly differentially expressed genes were reported for the Arabidopsis experiment, resulting in the missing horizontal band in the figure. The p-value for the statistical significance of the correlation and ${R}^{2}$ values for each time point are overlayed in the plots

ABA synthesis and signaling networks has been comprehensively discussed (Hauser et al. 2011). Hauser and colleagues list 147 *A. thaliana* genes that encode proteins involved in ABA responses, including transcription factors, kinases, ion channels, and signaling proteins (refer to Table S1 in Hauser et al. 2011). We investigated whether the expression of *B. napus* orthologs of these *A. thaliana* genes are also regulated by ABA in guard cells. Moreover, the *B. napus* orthologs of the ABA signaling *A. thaliana* genes were used to test for the enrichment of ABA responsive genes, as shown in Fig. 3.

A p-value (using the hypergeometric test) for the enrichment of the orthologs of the ABA signaling genes among differentially expressed genes (Tables S1 and S3) at 15 min was determined as P = $1.96\times {10}^{-19}$. For 60 min of ABA treatment, the p-value was $1.02\times {10}^{-51}$. To more thoroughly compare them and to estimate the effect size, we also examined chi-square and G tests and calculated the Cramer's V (which computes the correlation between two tables), evaluating the up-regulated and down-regulated genes separately (Tables S2 and S4).

**Fig. 4:**
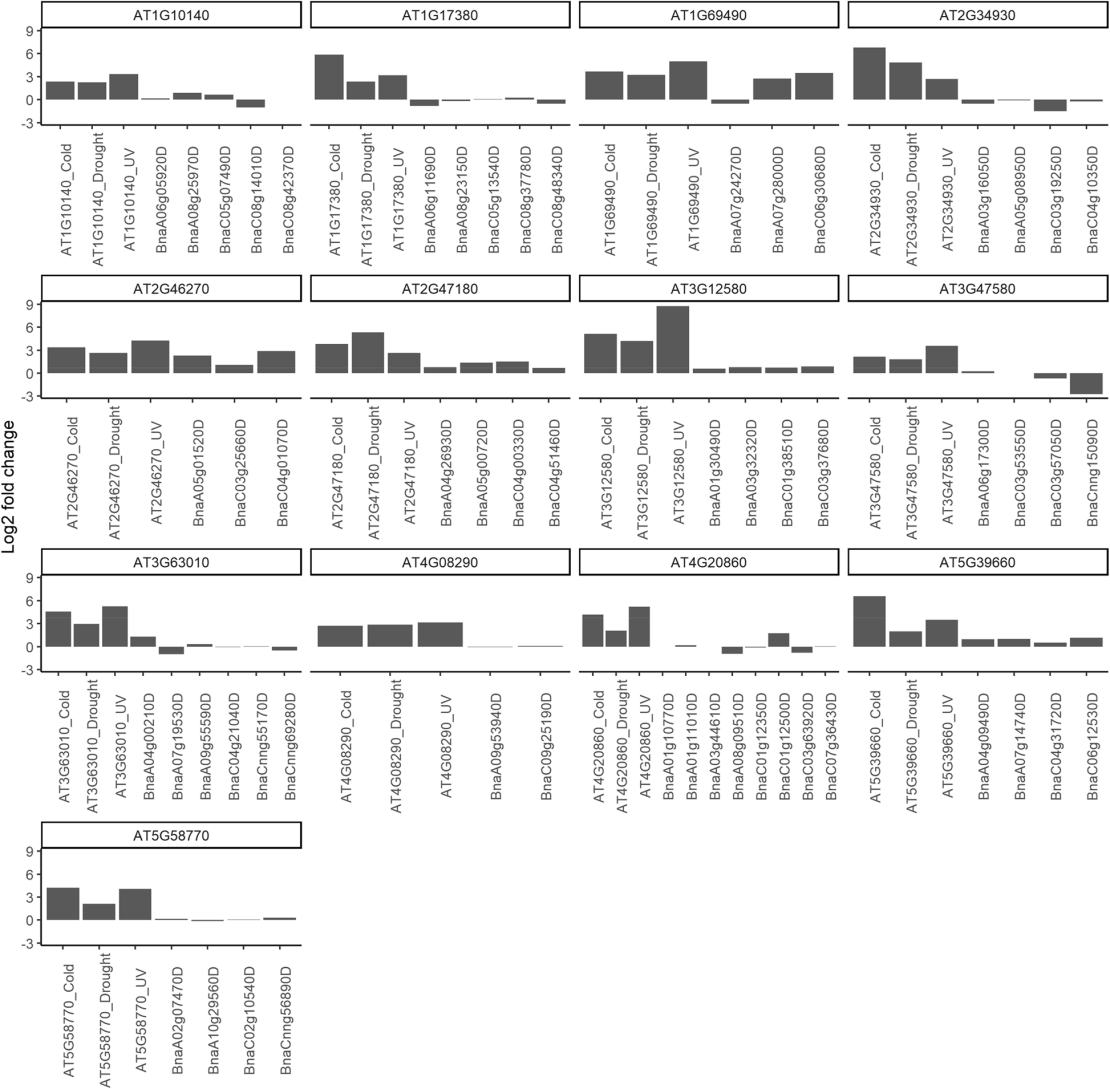
The differential expression of a number of stress markers from Kilian et al. (2007) with corresponding *B. napus* genes. y-axis shows log2 fold change in the expression level of the genes. The expression levels of Arabidopsis genes in response to cold, drought, and UV are shown. The expression level of the *B. napus* orthologs of each Arabidopsis gene is also shown

Even though the correlation between known ABA signaling pathway genes and differential expression is significant, the effect size (Cramer's V) is small at $0.048$ for 15 min and $0.058$ for 60 min. These results indicate that there is only a small core of genes in the ABA signaling pathway that are similarly regulated in response to ABA. To test whether this observation is unexpectedly low, we examined the statistical significance and effect size of the enrichment of ABA signaling genes reported for differentially expressed genes in *A. thaliana* (Tables S5 and S6). For genes significantly enriched for differential regulation in ABA signaling at both 15 min and 60 min of ABA treatment (p-value < $2\times {10}^{-16}$), we found that the correlation in *A. thaliana*, with a Cramer's V of $0.073,$ is only slightly better than that at 60 min for *B. napus* (0.058). We conclude that the ABA signaling network and its regulation in guard cells is largely conserved from *A. thaliana* to *B. napus* and that the regulation of the genes in this network is more pronounced at 60 min of treatment than at 15 min, consistent with an increased magnitude of the transcriptomic response. Because the signaling network is composed of various, additional processes such as post-translational modifications, protein binding, and transport, not all relevant genes are expected to be transcriptionally regulated. This consideration may be especially true for rapid responses, as in ABA-induced stomatal closure, and may explain the overall low effect size found for both conditions and both organisms. To compare the known stress responses with this ABA treatment, we analyzed the fold change of known stress markers taken from (Kilian et al. 2007) in Fig. 4.

## Regulatory interactions and the observed differential expression

**Fig. 5:**
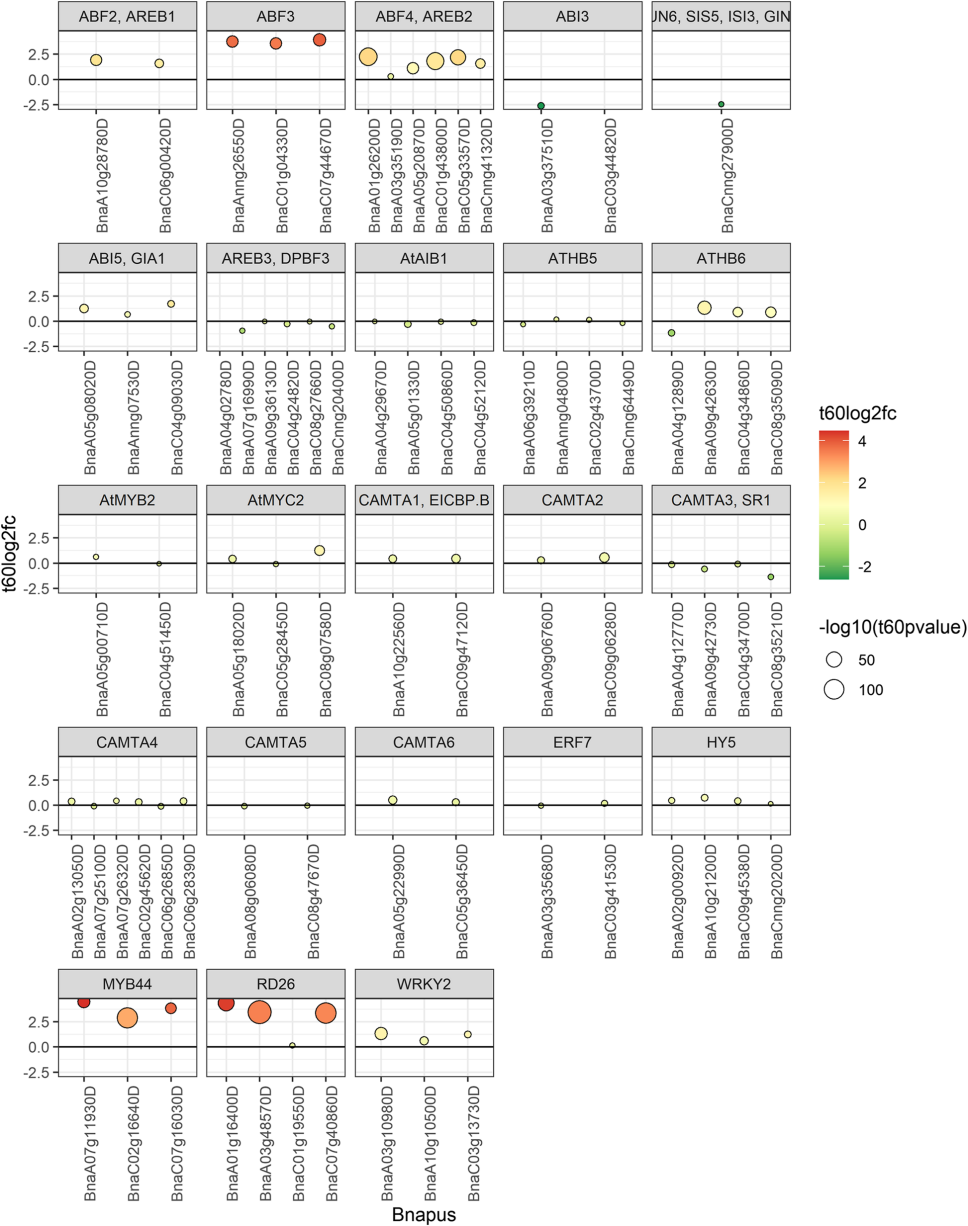
Fold changes of selected *B. napus* ABA related transcription factors after 60 min of ABA treatment

First, we plotted the differential expression of certain known ABA related transcription factors in Fig. 5. Notably, *ABF3*, *ABF4*, *MYB44*, and *RD26* were up-regulated. Next, we examined the observed differential expression in *B. napus* in reference to the known regulatory interaction network in *A. thaliana*. We used the ARGIS database, which collects known transcription factors and their targets from various *A. thaliana* studies (Davuluri et al. 2003). We examined the extent of agreement between our results and various high-throughput and low-throughput studies, and we also considered the implications for the utilization of the regulatory network during the ABA response. A particular external stimulus, like the application of ABA, may have relatively few direct protein targets. These targets convey the signal through a cascade of reactions such as protein binding, post-translational modifications like phosphorylation and dephosphorylation, and transcriptional factor-DNA binding, among others. The observed change in mRNA accumulation of any gene in response to the stimulus may result from a downstream regulatory relation like a transcription factor binding to a promoter element.

With potentially limited information to explain the changing expression levels of genes, one strategy is to consider the simple case differential gene expression occurring due to a transcription factor affecting the expression of its target. The theoretical effect of a known regulatory interaction on a target is inferred from the type of interaction (activation or repression) and the expression of the transcription factor (up-regulated or down-regulated). For example, if the expression of a transcription factor is up-regulated, and if this transcription factor is known to activate expression of a subset of target genes, we expect to observe the up-regulation of the target. In contrast, if an up-regulated transcription factor is known to repress the expression of its target, we expect to see the target down-regulated. We tabulated this proposed effect of the interaction versus the actual expression of the target under ABA treatment to see if a pattern of interaction might explain the expression of the target.

**Fig. 6:**
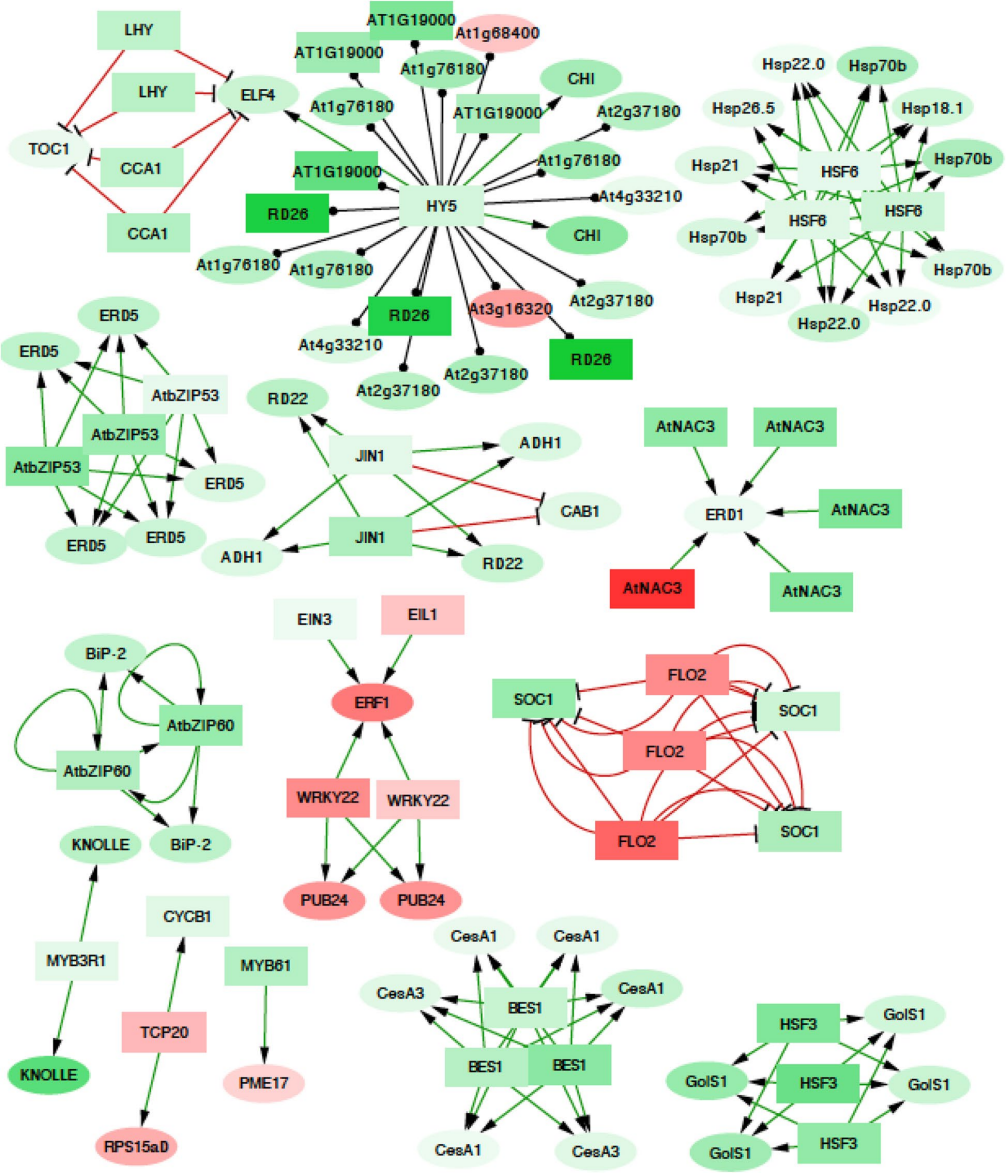
*B. napus* regulatory interactions compiled from the ARGIS low throughput studies (${\text{N}}<50$) among differentially expressed genes. The genes are colored for their direction for regulation, with green for up-regulated genes and red for down-regulated genes. The link ends denote the type of regulatory interactions, with pointed arrows, flat heads, and dots on the transcriptional target denoting activation, repression, and unknown interactions

We quantified the effect of the predicted regulation on the observed regulation with a $2\times 2$ contingency table summarized as the odds ratio, defined as the ratio of the fraction of up-regulated genes among those predicted to be up-regulated, divided by the fraction of up-regulated genes among those predicted to be down-regulated. An odds ratio larger than one implies that the estimate is predictive of the observed direction of regulation. However, the cross-tabulation does not show this effect when using all known interactions from the AGRIS database (Celli et al. 2015). Rather, the opposite is true (with an odds ratio of $0.46$). This result implies that many of these known interactions are not functional for the guard cell ABA response or that some subset of these studies may not agree with our results due to the nature of the experiments used to infer the regulatory interactions. For example, these interactions may not be present in all cell types. The accuracy and quality of interactome networks derived from high throughput systematic studies, and whether they are of similar confidence as individual studies, has been debated (Bader et al. 2004; Mrowka et al. 2001). Although the potential problems are better studied for protein–protein interactions, similar issues may arise in transcription factor studies. For this reason, it is reasonable to further evaluate our results in light of only the low throughput studies. Looking at regulatory interactions only from the low throughput studies reporting less than 50 *A. thaliana* interactions, we see a greater fraction of interactions where the direction of differential expression is consistent with the transcriptional interaction. With an odds ratio of 32.5, much larger than 1, and the test being highly statistically significant, the observed differential expression is consistent with the transcriptional network from low-throughput studies. This set of regulatory interactions from low-throughput studies among the differentially expressed genes of *B. napus* is visualized in Fig. 6.

## Proline biosynthesis is up-regulated

**Fig. 7:**
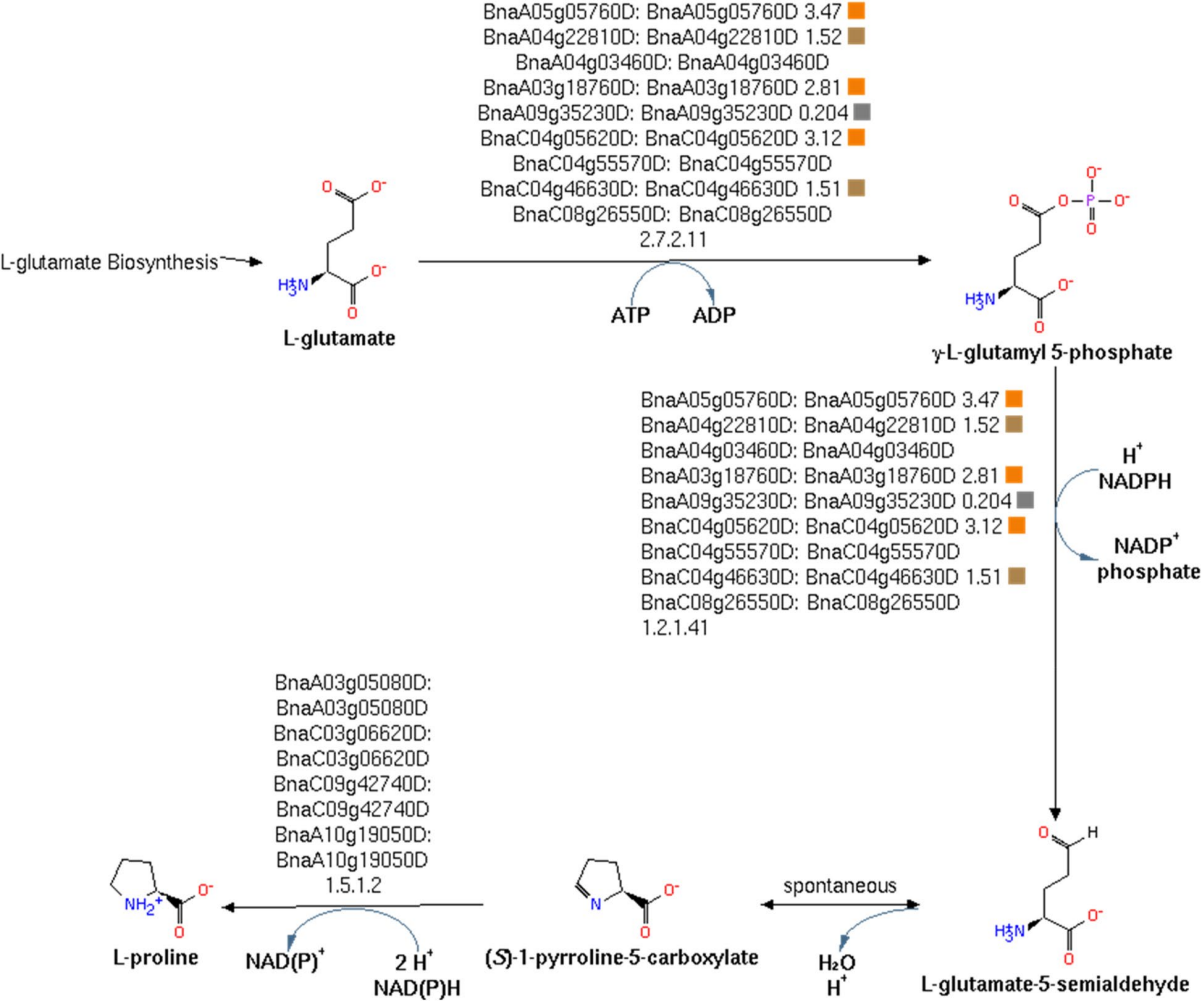
Proline biosynthesis is enriched for ABA responsive genes

We mapped pathways in *B. napus* from the corresponding Arabidopsis orthologs and the metabolic pathways in BioCyc (Caspi et al. 2016). As shown in Fig. 7, a number of putative enzymes catalyzing reactions for proline biosynthesis are up-regulated in response to ABA in Brassica guard cells. The role of proline during drought stress is well-known (Ghosh et al. 2022); and proline accumulation in response to drought has been observed in roots and leaves (Kesari et al. 2012; Sofo et al. 2004; Verslues & Bray 2006). Proline can have a number of roles, including increasing amounts of inorganic solutes characteristic of water loss (Samaras et al. 1995), acting as an osmolyte and preventing ROS accumulation (Liang et al. 2013; Wani et al. 2016). It has also been hypothesized that proline accumulation is a mechanism of storing energy to be released once the stress is relieved. Transgenically increasing the levels of delta-1-pyroline-5-carboxylate synthetase, which catalyzes the first step of proline synthesis in potato (Hmida-Sayari et al. 2005), petunia (Yamada et al. 2005) and tobacco (Kishor et al. 1995) was shown to confer drought resistance. The up-regulation of proline biosynthesis genes in guard cells by ABA may reflect the cell’s preparations for longer term drought responses.

## Differentially expressed targets of known ABA signaling transcription factors

Not all regulatory interactions may actually be functional in all physiological conditions. However, knowledge of important regulators of ABA responses can be used to predict potential targets for further enquiry (Table S8). Using the high-confidence (i.e., low throughput) set of regulatory interactions, we selected sets of interactions for which the transcription factors are known ABA signaling molecules and both the regulatory factor and target are differentially expressed. We found 11 transcription factor-target relationships that satisfied these criteria (Table S8). This result suggests candidate genes among the targets that are likely to be involved in ABA signaling. For instance, *RD22* (*RESPONSIVE TO DESICCATION 22*) and *RD26* (*RESPONSIVE TO DESICCATION 26*) have known roles in the response to water deprivation. AT1G76180 (*EARLY RESPONSE TO DEHYDRATION 14, ERD14*) is a Ca^2+^ binding protein involved in the early response to dehydration and cold (Kiyosue et al. 1994). *ADH1* is known to be regulated by both dehydration and hypoxia, *CHI* is involved in the response to UV light, and *ELF4* regulates flowering time.

## Regulation inferred from transcription factor binding sites

The effect of a transcription factor on the expression of its target involves the binding of the transcription factor to a cis-regulatory element in the target gene's promoter region. A transcription factor, or a class of transcription factors, may recognize a specific polynucleotide sequence (Franco-Zorrilla et al. 2014). These binding sites in the promoter regions of *B. napus* genes could provide indications of putative regulatory interactions between *B. napus* transcription factors and their targets. This information may provide a finer view of the regulatory network than simply mapping *A. thaliana* interactions to all the corresponding *B. napus* orthologs.

To determine candidate transcription factors responsible for the differential expression in response to ABA, we searched for known binding sites defined from AtTFDB (Davuluri et al. 2003) in the promoter regions of *B. napus* genes. For each known Arabidopsis transcription factor binding site sequence in our dataset, we calculated the enrichment of the occurrence of the sequence in the promoters of the up-regulated (Table S9) and down-regulated (Table S10) genes versus non-changing genes, with statistical significance provided by the hypergeometric *p*-value adjusted for multiple correction. This analysis revealed that binding sites over-represented in the promoter regions of *B. napus* genes regulated by ABA (see Tables S9 and S10) are also enriched in the promoter regions of *A. thaliana* guard cell ABA responsive genes (Wang et al. 2011). Specifically, the putative ABA responsive element (ABRE) targets that are regulated in an ABA-dependent manner in our study are also up-regulated during drought and cold stress in an ABA-independent manner in other datasets (Agarwal & Jha 2010; Narusaka et al. 2003).

There are 170 distinct *B. napus* transcription factors whose binding sites are enriched in the promoter regions of the up-regulated genes (Table S8). Out of these 170 genes, 29 genes are themselves up-regulated (17%) as opposed to only 1,944 up-regulated genes (with an FDR of less than 5%) out of the total 101,040 genes (1.9%), giving a statistically significant hypergeometric *p*-value of $4.11\times {10}^{-20}$ and an effect size of 0.045 in terms of Cramer’s V. Out of the 170 *B. napus* transcription factors, 23 are known members of the ABA signaling pathway (13.5%), whereas only 435 out of the total of 101,040 genes (0.4%) are members of the ABA pathway. This leads to a statistically significant hypergeometric p-value of $4.86\times {10}^{-29}$ with a Cramer’s V of 0.082.

## Conclusions

ABA-mediated signaling plays a major role in plant responses to a number of stresses, both biotic and abiotic (Seo & Koshiba 2002; Zhu 2002). The experimental design simplifies the investigation of drought signaling occurring in plants by focusing on relatively rapid ABA-triggered transcriptomic response in a single cell type, namely *Brassica* guard cells. In addition to statistical analysis of the gene expression data, we combined the expression data with known information about metabolic pathways, gene regulatory interactions involving transcription factors and DNA regulatory elements, and evolutionary comparisons to Arabidopsis to highlight conserved mechanisms involved in ABA responses in this cell type. We found qualitatively similar gene expression responses as well as unique gene expression responses at 15 and 60 min of ABA application. The extent of regulation increases from 15 to 60 min, consistent with a mechanism in which the transcription rate is constant with negligible transcript degradation. Only a few genes show statistically significant regulation with a different dynamic pattern (Table 2).

Comparisons with Arabidopsis show considerable divergence of gene expression in paralogous gene families, but the level of correlation within families is still high. While statistically correlated, we found many differences in the measured guard cell ABA response between *B. napus* and *A. thaliana*. Despite the low statistical power, and hence a smaller number of genes identified at 15 min of ABA treatment, these genes showed a much higher concordance with the Arabidopsis response, with only BnaC02g37590D (corresponding to AT3G28910 or ATMYB30) and BnaC04g28450D (corresponding to AT3G51910 or AT-HSFA7A) showing regulation in the opposite direction. These two genes were up-regulated in *B. napus*, while their Arabidopsis orthologs were down-regulated. Furthermore, we show that the non-coding DNA regulatory elements have diverged within paralogous families, and evolutionary divergence has affected the expression of their target genes.

Among the metabolic pathways, proline synthesis was found to be up-regulated, consistent with the previous studies. Interestingly, data collected on *aba2-1* subjected to low water potential (Sharma & Verslues 2010) show that proline accumulation is only partially impaired in the mutant, suggesting other regulatory processes are at work. However, when ABA was exogenously applied, proline accumulation was observed back to its expected level, indicating that ABA still directly participates to proline accumulation to some extent. We, therefore, cannot exclude that we are observing the joint effect of ABA application and changes in osmotic pressure, although the role of ABA – even if partial – is confirmed here.

A statistically significant part of the ABA signaling pathway is up-regulated, but most genes in the pathway do not change their expression; and these results were similar for both Arabidopsis and Brassica. We found that regulatory interactions reported in individual small-scale studies in Arabidopsis were more consistent with the observed *B. napus* gene expression profiles than from large-scale screens. We selected these interactions related to the more defined studies to generate the regulatory interaction graph shown in Fig. 7. The regulatory interactions are likely to be actively involved with the ABA response.

## Materials and Methods

### Plant material and growth conditions

All *Brassica napus* plants used in this study were from the double-haploid line DH12075. Brassica seeds were sown on Sunshine Redi-earth Plug & Seedling Mix (Sun Gro Horticulture, Canada) and then stratified for at least 2 d at 4 °C. The plants were grown at 60% relative humidity in 16 h light at 21 °C and in 8 h dark at 18 °C.

## Isolation of guard cell protoplasts

Brassica leaves (~ 70 g) 5–7 weeks old were excised and their central veins removed before blending for 3 × 1 min with a Waring blender in cold water. This first step aims at eliminating mesophyll cells while guard cells are retained in epidermal fragments (See Fig. S1A). After filtering through a nylon mesh (pore size 200 µm) to remove all remaining mesophyll cells, the epidermal fragments were washed thoroughly with water and transferred to a flask containing 100 mL of 0.7% Cellulase R-10 (Yakult Pharmaceutical, Tokyo, Japan), 0.05% Macerozyme R-10 (Yakult), 0.10% polyvinylpyrrolidone 40, 0.25% BSA, 0.5 mM ascorbic acid, and 55% basic medium (0.5 mM CaCl_2_, 0.5 mM MgCl_2_, 5 mM MES hydrate, 0.5 mM ascorbic acid, 10 µM KH_2_PO_4_, 0.53 M D-sorbitol, pH 5.5). The epidermal fragments were incubated in a shaking water bath (175 RPM) at 22 °C for 40–50 min in the dark to digest all epidermal and mesophyll cells. To adjust the osmolality in preparation for the second enzyme digestion, 150 mL of basic medium were added, and the epidermal fragments were incubated for an additional 10 min prior to being collected using a nylon mesh (pore size 200 µm) and washed two times with basic medium. The epidermal fragments were then transferred into a flask containing 50 mL of 1.1% Cellulase RS (Yakult), 0.0075% Pectolyase Y-23 (Duchefa Biochemie, Haarlem, Netherlands), 0.25% BSA, 0.5 mM ascorbic acid, and 100% basic medium. After incubating in a shaking water bath (100 RPM) at 22 °C for 1–1½ h in the dark, the solution containing free guard cell protoplasts was filtered through a single layer of nylon mesh (pore size 20 µm). Basic medium was also poured through the mesh to rinse the epidermal fragments for a total volume of 400 mL. The protoplast solution was centrifuged at 350* g* for 5 min, after which the supernatant was removed. The pellet was resuspended in a small volume of basic medium and then layered carefully on top of an equal volume of gradient solution containing 35% basic medium and 65% Histopaque (Sigma-Aldrich, St. Louis, MO, USA). Following centrifugation at 430* g* for 5 min, the guard cell protoplasts at the interface of the two solutions were isolated. Guard cell number and purity were determined using a hemacytometer. Protoplast preparations with a purity of ~ 99% were kept at 4 °C during the procedure, until used for subsequent experiments.

Next, guard cell protoplasts were subjected to a 10 µM ABA treatment (EtOH was added to the mock-treated sample at an equal proportion) and kept under light at room temperature for 15 min and 60 min. The samples were flash-frozen in liquid nitrogen before being stored at -80 °C until enough material had been collected. Total RNA from 3 independent pools of 3 to 5 preparations each was extracted using the RNeasy Plant Mini Kit (Qiagen) and prepared for sequencing.

## Analysis of differential expression

All the RNAseq reads (100 bp single ended) were aligned to the Darmor genome with TopHat2 (Kim et al. 2013). Reads mapping to genes were counted with HTSeq (Anders et al. 2015). Multi-mapped reads were discarded by HTSeq due to low mapping quality. This lowers the statistical power to detect differential expression for genes with many close paralogs because sequencing reads may align to different paralogs. However, removing these ambiguously mapped reads means that we are confident that we are correctly differentiating the different paralogs and differentially expressed genes are correctly called. Differential expression was calculated with DESeq2 (Love et al. 2014).

Batch effect on replicates was noted and accounted for by including the replicate information in the linear model design matrix. The expected expression level of a gene $i$ in sample $j$ was modeled as

$${q}_{ij}={s}_{j}\sum^{r}{x}_{jr}{\beta }_{ri},$$

where ${s}_{j}$ is the sample normalization, ${x}_{jr}$ is the effect $r$ on sample $j$ and ${\beta }_{ir}$ is the effect $r$ on gene $i$. Specifically in our case, if we arrage our samples blockwise with time as t = 0 (Replicate 1), t = 0 (Replicate 2), t = 0 (Replicate 3), t = 15 (Replicate 1), t = 15 (Replicate 2), t = 15 (Replicate 3), t = 60 (Replicate 1), t = 60 (Replicate 2), t = 60 (Replicate 3), the design matrix $x$ is

$$x=\left[\begin{array}{ccccccccc}1& 1& 1& 0& 0& 0& 0& 0& 0\\ 0& 0& 0& 1& 1& 1& 0& 0& 0\\ 0& 0& 0& 0& 0& 0& 1& 1& 1\\ 1& 0& 0& 1& 0& 0& 1& 0& 0\\ 0& 1& 0& 0& 1& 0& 0& 1& 0\\ 0& 0& 1& 0& 0& 1& 0& 0& 1\end{array}\right],$$

where the first 3 rows of the design matrix correspond to the effect of the 3 time points, while the latter 3 rows correspond to the effect of the 3 replicates. This design matrix separates the effects of the conditions and replicates that we observe in Fig. 1.

The actual read counts ${K}_{ij}$ for gene $i$ and sample $j$ are assumed to be sampled from a negative binomial distribution with the expected expressions ${q}_{ij}$ and a gene-specific dispersion ${\alpha }_{i}$. The corresponding probability mass function of the read counts is

$${\text{Pr}}({K}_{ij}=k)=\frac{\Gamma (k+1/{\alpha }_{i})}{k!\Gamma (1/{\alpha }_{i})}{\left(\frac{{q}_{ij}}{{q}_{ij}+1/{\alpha }_{i}}\right)}^{k}{\left(\frac{1}{1+{q}_{ij}{\alpha }_{i}}\right)}^{1/{\alpha }_{i}}.$$

The dispersion for a gene ${\alpha }_{i}$ is a shrinkage estimate based on all the observed genes that decreases with increasing mean read counts, as shown in Fig. S1. The p-values for differential expression were obtained by the Wald's test (Wald 1943). Genome wide significance was evaluated by adjusting for multiple testing using the Benjamini–Hochberg correction (Benjamini & Hochberg 1995) and an independent filtering step based on the mean expression rate. An adjusted p-value cutoff corresponding to 0.05 FDR was used to call a gene differentially expressed in all downstream analyses.

## Evaluating the significance of the binding site gain/loss

We use a generalized linear model to evaluate the significance of the loss or gain of a binding site on the differential expression. For a gene $i$, let us denote the fold change in expression at $60$ minutes of ABA tretment to $0$ minutes of ABA treatment as ${\beta }_{i,60}/{\beta }_{i,0}$. Also, for a particular binding site sequence, let the number of binding site sequences occuring in the promoter region be denoted as ${n}_{i,{\text{BSS}}}$ and its mapped *Arabidopsis thaliana* ortholog be coded as the categorical variable ${{\text{O}}}_{i}$. If there are a total of $M$ *A. thaliana* genes, then ${{\text{O}}}_{i}$ is an *M*x1 vector of all zeros except one $1$ for the corresponding Arabidopsis gene. We model the log2-fold ratio as

$${\text{log}}2\left(\frac{{\beta }_{i,60}}{{\beta }_{i,0}}\right)={\beta }_{{\text{BSS}}}{n}_{i,{\text{BSS}}}+{\beta }_{{\text{Orth}}}{{\text{O}}}_{i}.$$

The parameter ${\beta }_{{\text{Orth}}}$ is simply a vector of the mean ${\text{log}}2$-fold ratio for each gene family, where a gene family are all the *Brassica napus* genes corresponding to the same *Arabidopsis thaliana* gene. The parameter ${\beta }_{{\text{BSS}}}$ captures the effect of the presence of a binding site sequence on the fold change after correcting for the common ancestry of the genes within a gene family.

We do not necessarily expect the ${\text{log}}2$ fold changes to be linearly dependent on the binding site sequence presence. However, a significant non-zero value of ${\beta }_{{\text{BSS}}}$ should signal the dependence of the fold change on the presence of binding site sequence, in a statistical sense. The statistical significance is evaluated as the p-value of the F-test testing for the null hypothesis of ${\beta }_{{\text{BSS}}}=0$ and the alternative hypothesis of ${\beta }_{{\text{BSS}}}\ne 0$. Since the diagnostic statistics and F-tests for the high-dimensional categorical predictor parameter ${\beta }_{{\text{Orth}}}$ were not required, all parameters were estimated by modeling the categorical models as fixed effects using the lfe R package (Gaure 2013).

## Nucleotide substitution rates

Amino acid sequences of the translated cDNAs were aligned using ClustalW2 (Larkin et al. 2007). The aligned protein sequences were used to align the nucleotide sequences using transAlign (Bininda-Emonds 2005). The synonymous and non-synonymous nucleotide substitution rates were calculated in the seqinr R package (Charif & Lobry 2007) using the model of Li (Li 1993). Any values of ${k}_{s}$ and ${k}_{a}$ greater than 2 were discared as missing values for subsequent analysis assuming that these might be incorrect ortholog assignments or alignments because we do not expect to observe substitution rates this high across the length of any gene.

## Supplementary Information

Supplementary file 1: **Figure S1 - Figure S5.**

Supplementary file 2: **Table S1 - Table S10.**

## Data Availability

Data used for this study are available at https://www.ncbi.nlm.nih.gov/geo/query/acc.cgi?acc=GSE254116.

## Abbreviations

ABA: Abscisic acid
ABF: ABRE-binding factor
ABRE: ABA responsive element
ADH: Alcohol dehydrogenase
AGRIS: Arabidopsis gene regulatory information server
AtTFDB: Arabidopsis transcription factor database
BSA: Bovine serum albumin
ELF: Early flowering
ERD: Early response to dehydration
CHI: Chitinase
FAB1C: Phosphatidylinositol-3-phosphate 5-kinase
FDR: False discovery rate
HTSeq: High-throughput sequence analysis in Python
MAPKKK: Mitogen-activated protein kinase kinase kinase
MES: 2-(N-morpholino)ethanesulfonic acid
RD: Responsive to desiccation
ROS: Reactive oxygen species
SnRK2: SNF1-related protein kinase 2
