# Supplementary material for: Transcriptomic dynamics of ABA response in Brassica napus guard cells
Source: Stress Biol. 2024 Oct 14;4(1):43. doi: 10.1007/s44154-024-00169-7 (PMC11473748; doi:10.1007/s44154-024-00169-7)
Supplement: Supplementary file 1 — Figure S1 - Figure S5. [file 44154_2024_169_MOESM1_ESM.docx]

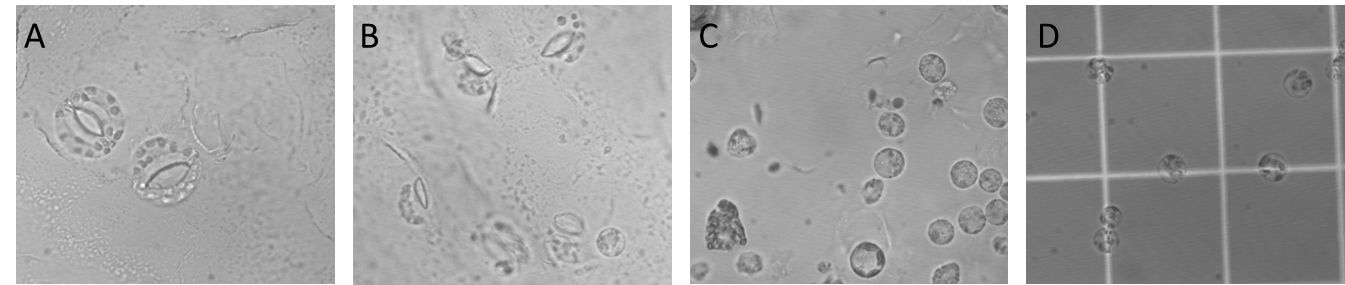


**Fig. S1** Key steps in the purification of isolated guard cells from Brassica napus leaves. Epidermal fragments (A) were thoroughly washed before being subjected to enzymatic digestion of the cell wall to release protoplasts (B). Guard cells protoplasts with minor epidermal cells contamination were then retrieved (C) and purified by centrifugation for subsequent treatment and analysis (D).


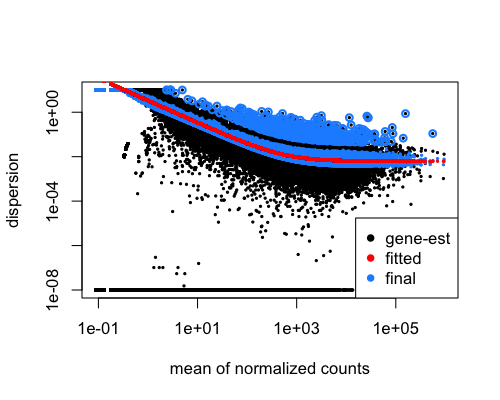


**Fig. S2** Estimate of the dispersion (coefficient of variation) in the noise model for read counts plotted versus the mean read count of a gene. The downward slope implies a negative binomial distribution rather than the Poisson model where the coefficient of variation 1 for all mean values.


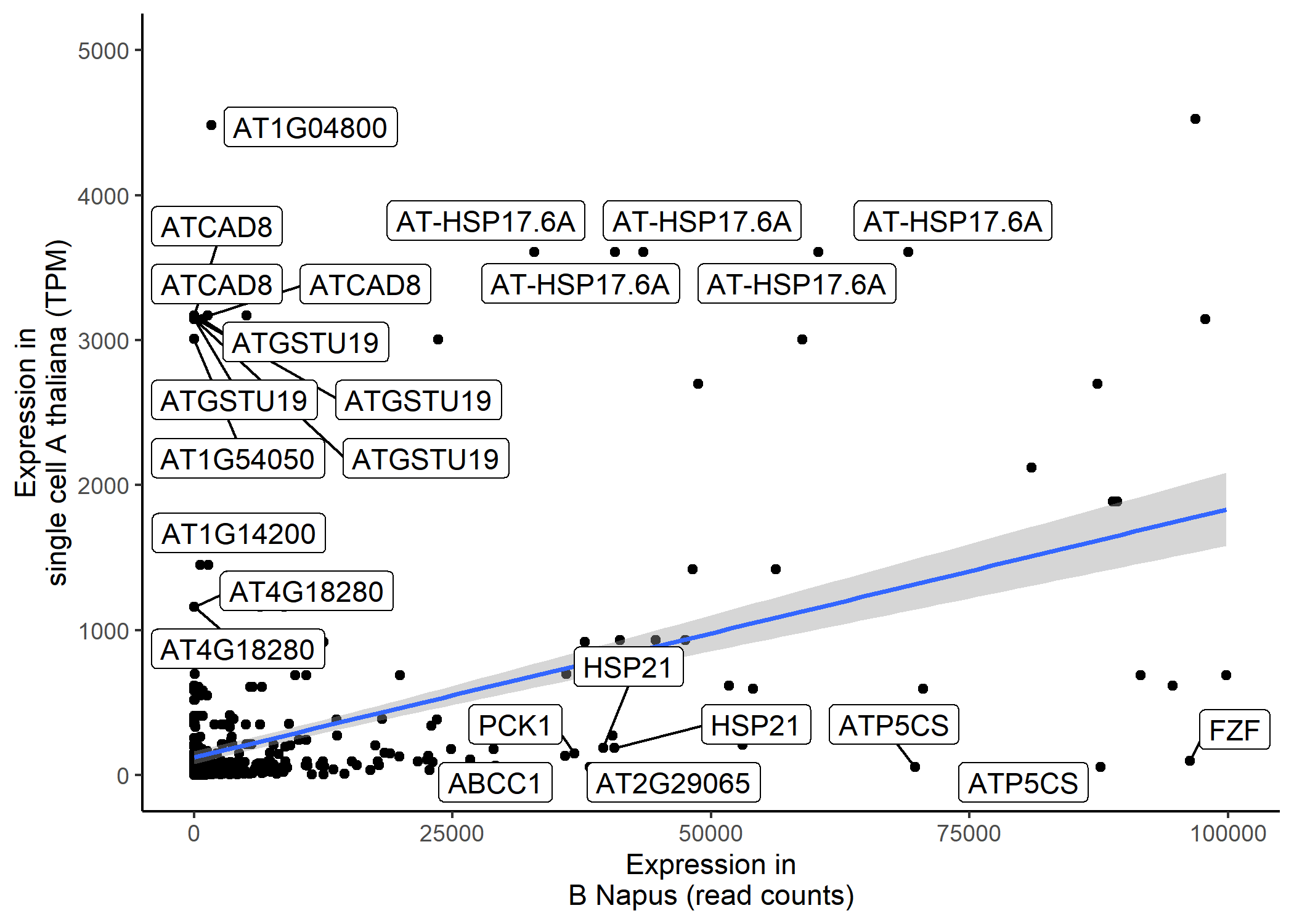


**Fig. S3** Comparison of the expression in sf6 cluster from the single cell paper (representing guard cells) from single-cell stomata data from Lopez-Anido et al. (2021), with average expression (before and after ABA treatment). The overall statistical significance of the linear correlation is a p-value of 10-15. The genes with more divergent expression between the two datasets are marked. Notably, heat shock proteins and proline synthesis pathway genes have divergent expression Gene expression values at the two times are correlated. We plot the log2 fold changes at 15 minutes vs. 60 minutes of ABA treatment for all genes that are significantly regulated (< 5% FDR).


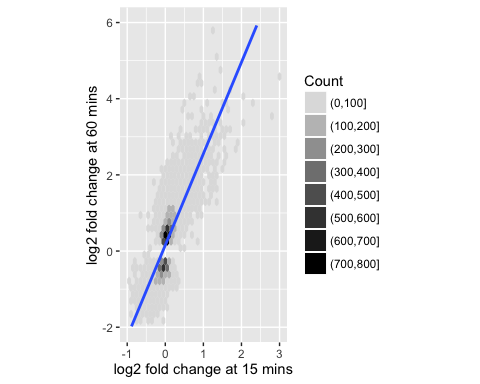


**Fig. S4** Gene expression values at the two times are correlated. We plot the log2 fold changes at 15 minutes vs. 60 minutes of ABA treatment for all genes that are significantly regulated (< 5% FDR) at either time. Each cell in the plot shows the density of genes with a particular combination of log2 fold changes at each time point. The straight line is the linear best fit. Genes that are not significantly differentially expressed at either time are excluded; the best fit estimate reflects stable regulation rather than background variation.


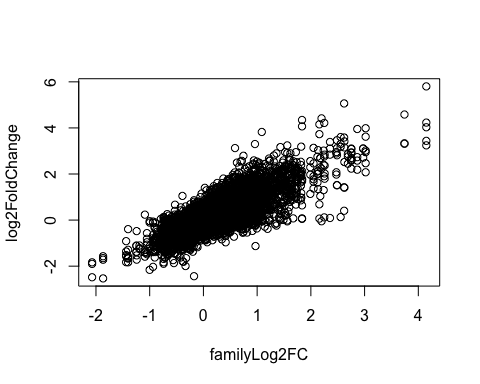
(A)


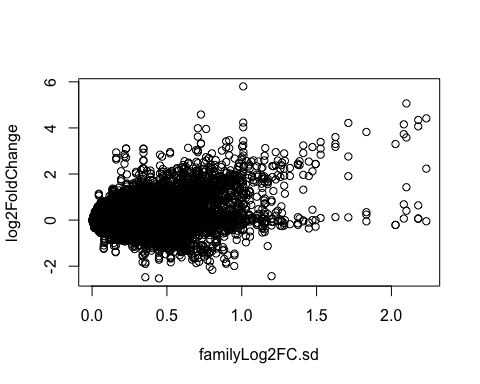


**(B)**

**Fig. S5** (A) Plot of the log2 fold change at 60 minutes of a gene against the family-wise log2 fold change, where family wise log2 fold change is defined as the mean log2 fold change of the group of paralogous genes corresponding to the same A. thaliana gene. (B) Plot of the standard deviation of the family log2 fold change vs. the gene log2 fold change.
