# Supplementary material for: Transcriptomic dynamics of ABA response in Brassica napus guard cells
Source: Stress Biol. 2024 Oct 14;4(1):43. doi: 10.1007/s44154-024-00169-7 (PMC11473748; doi:10.1007/s44154-024-00169-7)
Supplement: Supplementary file 2 — Table S1 - Table S10. [file 44154_2024_169_MOESM2_ESM.docx]

**SUPPEMENTAL TABLES**

**Table S1.** ABA related genes from Hauser et al 2001, that are highly regulated in our experiment, i.e., had an absolute fold change of more than 2, and fdr of less than .01.

| Brassica napus gene | Arabidopsis thaliana ortholog | t60 adjusted pvalue | t60 log2fc | Arabidopsis thaliana common name |
| --- | --- | --- | --- | --- |
| BnaA01g03110D | AT4G33950 | 4.35e-28 | 2.021 | ATOST1 |
| BnaA01g15250D | AT4G26080 | 5.86e-25 | 1.296 | ABI1 |
| BnaA01g16400D | AT4G27410 | 4.69e-52 | 4.339 | ANAC072 |
| BnaA01g26200D | AT3G19290 | 2.26e-74 | 2.219 | ABF4 |
| BnaA01g37370D | AT3G11410 | 2.38e-22 | 5.981 | AHG3 |
| BnaA02g14680D | AT1G69270 | 1.37e-34 | 1.35 | AtRPK1 |
| BnaA03g04440D | AT5G13630 | 6.12e-42 | 2.051 | ABAR |
| BnaA03g10980D | AT5G56270 | 8.52e-26 | 1.341 | ATWRKY2 |
| BnaA03g17720D | AT2G38310 | 7.48e-30 | -1.417 | PYL4 |
| BnaA03g41510D | AT3G51860 | 7.92e-15 | 1.013 | ATCAX3 |
| BnaA03g43490D | AT4G17970 | 2.36e-58 | 2.179 | ALMT12 |
| BnaA03g43960D | AT4G19230 | 9.31e-16 | 1.315 | CYP707A1 |
| BnaA03g48570D | AT4G27410 | 4.30e-145 | 3.469 | ANAC072 |
| BnaA04g17140D | AT2G29940 | 6.43e-25 | 1.55 | ABCG31 |
| BnaA04g29300D | AT2G40330 | 9.35e-11 | -1.881 | PYL6 |
| BnaA05g05340D | AT2G46070 | 3.29e-31 | 1.212 | ATMPK12 |
| BnaA05g05420D | AT2G40330 | 1.36e-08 | -5.021 | PYL6 |
| BnaA05g08020D | AT2G36270 | 3.17e-07 | 1.262 | ABI5 |
| BnaA05g12650D | AT2G29380 | 2.41e-03 | 2.445 | HAI3 |
| BnaA05g20870D | AT3G19290 | 1.36e-20 | 1.111 | ABF4 |
| BnaA05g27660D | AT3G11410 | 3.16e-27 | 5.307 | AHG3 |
| BnaA06g40360D | AT5G46790 | 6.77e-10 | -1.148 | PYL1 |
| BnaA07g11930D | AT5G67300 | 9.72e-23 | 4.475 | ATMYB44 |
| BnaA07g12170D | AT5G67030 | 1.53e-17 | 1.22 | ABA1 |
| BnaA07g24330D | AT1G69260 | 5.02e-40 | 4.785 | AFP1 |
| BnaA07g29760D | AT1G71960 | 7.41e-04 | 2.197 | ABCG25 |
| BnaA07g30430D | AT1G72770 | 3.57e-08 | 2.077 | AtHAB1 |
| BnaA08g23040D | AT1G17550 | 6.55e-26 | 1.435 | AtHAB2 |
| BnaA09g07610D | AT5G67030 | 2.66e-19 | 4.733 | ABA1 |
| BnaA09g20620D | AT4G04020 | 4.02e-45 | 1.706 | FIB |
| BnaA09g26270D | AT1G30270 | 5.99e-03 | -1.025 | ATCIPK23 |
| BnaA09g40690D | AT2G26040 | 8.62e-07 | -1.045 | PYL2 |
| BnaA09g42630D | AT2G22430 | 5.04e-32 | 1.319 | ATHB6 |
| BnaA09g49440D | AT1G07430 | 5.70e-78 | 3.646 | AIP1 |
| BnaA10g00540D | AT1G01360 | 4.35e-34 | 1.186 | PYL9 |
| BnaA10g04840D | AT1G07430 | 2.64e-38 | 4.253 | AIP1 |
| BnaA10g11080D | AT5G57050 | 1.06e-57 | 2.118 | ABI2 |
| BnaA10g12140D | AT5G59220 | 7.24e-31 | 5.169 | HAI1 |
| BnaA10g24990D | AT5G05440 | 8.53e-11 | -1.44 | PYL5 |
| BnaA10g28780D | AT1G45249 | 2.37e-17 | 1.931 | ABF2 |
| BnaAnng00100D | AT5G01820 | 1.35e-31 | 2.256 | ATCIPK14 |
| BnaAnng07990D | AT5G47910 | 5.96e-03 | -1.025 | ATRBOHD |
| BnaAnng13200D | AT2G38310 | 3.97e-07 | -2.349 | PYL4 |
| BnaAnng26550D | AT4G34000 | 4.47e-20 | 3.754 | ABF3 |
| BnaAnng35310D | AT4G17870 | 2.15e-05 | -1.333 | PYR1 |
| BnaAnng40650D | AT5G05440 | 9.19e-06 | -1.962 | PYL5 |
| BnaC01g04330D | AT4G34000 | 8.99e-20 | 3.563 | ABF3 |
| BnaC01g04370D | AT4G33950 | 1.12e-19 | 1.462 | ATOST1 |
| BnaC01g18020D | AT4G26080 | 1.70e-23 | 1.249 | ABI1 |
| BnaC01g43800D | AT3G19290 | 3.10e-66 | 1.792 | ABF4 |
| BnaC02g16640D | AT5G67300 | 3.80e-104 | 2.889 | ATMYB44 |
| BnaC03g02130D | AT5G05440 | 1.24e-12 | -1.834 | PYL5 |
| BnaC03g05980D | AT5G13630 | 7.59e-11 | 2.265 | ABAR |
| BnaC03g21240D | AT2G38310 | 1.13e-36 | -1.937 | PYL4 |
| BnaC03g22610D | AT2G40330 | 2.59e-07 | -2.898 | PYL6 |
| BnaC04g04740D | AT2G46070 | 8.68e-26 | 1.003 | ATMPK12 |
| BnaC04g04830D | AT2G40330 | 1.81e-04 | -3.055 | PYL6 |
| BnaC04g07010D | AT2G38310 | 6.22e-07 | -2.147 | PYL4 |
| BnaC04g40750D | AT2G29940 | 6.45e-23 | 1.363 | ABCG31 |
| BnaC04g47050D | AT2G40330 | 2.30e-31 | -1.962 | PYL6 |
| BnaC04g50710D | AT2G46070 | 3.46e-05 | 1.218 | ATMPK12 |
| BnaC05g05180D | AT1G07430 | 6.94e-56 | 6.552 | AIP1 |
| BnaC05g33570D | AT3G19290 | 8.76e-49 | 2.188 | ABF4 |
| BnaC05g41830D | AT3G11410 | 3.32e-27 | 5.501 | AHG3 |
| BnaC06g00420D | AT1G45249 | 5.99e-07 | 1.581 | ABF2 |
| BnaC06g25430D | AT1G69260 | 6.24e-46 | 5.283 | AFP1 |
| BnaC06g32970D | AT1G71960 | 1.63e-22 | 3.266 | ABCG25 |
| BnaC06g33910D | AT1G72770 | 5.34e-08 | 1.774 | AtHAB1 |
| BnaC07g16030D | AT5G67300 | 8.18e-17 | 3.839 | ATMYB44 |
| BnaC07g34970D | AT4G17970 | 1.14e-13 | 2.038 | ALMT12 |
| BnaC07g40100D | AT4G26080 | 1.09e-19 | 1.035 | ABI1 |
| BnaC07g40860D | AT4G27410 | 1.04e-109 | 3.379 | ANAC072 |
| BnaC07g44670D | AT4G34000 | 2.72e-25 | 3.931 | ABF3 |
| BnaC08g06690D | AT1G35720 | 2.95e-09 | 1.133 | ANN1 |
| BnaC08g07580D | AT1G32640 | 1.88e-11 | 1.251 | ATMYC2 |
| BnaC08g11070D | AT4G17870 | 3.33e-23 | -1.315 | PYR1 |
| BnaC08g12100D | AT4G26080 | 1.08e-18 | 1.129 | ABI1 |
| BnaC08g17440D | AT1G17550 | 6.41e-20 | 1.554 | AtHAB2 |
| BnaC09g07550D | AT5G67030 | 2.65e-10 | 3.434 | ABA1 |
| BnaC09g22960D | AT4G04020 | 3.10e-29 | 1.295 | FIB |
| BnaC09g34350D | AT5G59220 | 1.29e-28 | 4.674 | HAI1 |
| BnaC09g49910D | AT5G05440 | 1.39e-05 | -2.212 | PYL5 |
| BnaC09g53650D | AT5G57050 | 1.80e-64 | 1.951 | ABI2 |
| BnaCnng26240D | AT1G69270 | 3.36e-28 | 1.207 | AtRPK1 |
| BnaCnng41320D | AT3G19290 | 5.66e-10 | 1.565 | ABF4 |
| BnaCnng74600D | AT4G04020 | 1.29e-39 | 1.479 | FIB |

**Table S2.** Cross-tabulation of membership in the known ABA signaling network against differential expression at 15 minutes of ABA treatment in B. napus.

|  | Down-regulated in Brassica (15 min.) | Insignificantly regulated in Brassica (15 min.) | Up-regulated in Brassica (15 min.) |
| --- | --- | --- | --- |
| Gene not part of the ABA signaling network | 68 | 100153 | 385 |
| Gene part of the ABA signaling network | 3 | 410 | 21 |

**Table S3**. Tests of statistical significance and the estimated effect size for the association of the known ABA signaling genes and those differentially expressed under 15 minutes of ABA treatment in B. napus.

| Test of association | Statistic | Degrees of freedom | P value | Effect size measure | Effect size |
| --- | --- | --- | --- | --- | --- |
| G-test | 76.50 | 2 | < 2.22e-16 | Contingency coefficient | 0.05 |
| Pearson's $\chi^{2}$ test | 238.58 | 2 | < 2.22e-16 | Cramer's V | 0.05 |

**Table *S*4.** Cross-tabulation of membership in the known ABA signaling network against. differential expression at 15 minutes of ABA treatment in B. napus.

|  | Down-regulated in Brassica (60 min.) | Insignificantly regulated in Brassica (60 min.) | Up-regulated in Brassica (60 min.) |
| --- | --- | --- | --- |
| Gene not part of the ABA signaling network | 4293 | 88857 | 7456 |
| Gene part of the ABA signaling network | 53 | 258 | 123 |

**Table *S*5.** Tests of statistical significance and the estimated effect size for the association of the known ABA signaling genes and those differentially expressed under 60 minutes of ABA treatment in B. napus.

| Test of association | Statistic | Degrees of freedom | P value | Effect size measure | Effect size |
| --- | --- | --- | --- | --- | --- |
| G-test | 235.59 | 2 | < 2.22e-16 | Contingency coefficient | 0.06 |
| Pearson's $\chi^{2}$ test | 356.64 | 2 | < 2.22e-16 | Cramer's V | 0.06 |

**Table S6.** Cross-tabulation of membership in the known ABA signaling network vs. differential expression in A. thaliana due to ABA treatment.

|  | Down-regulated in Arabidopsis | Statistically insignificant regulation in Arabidopsis | Up-regulated in Arabidopsis |
| --- | --- | --- | --- |
| Gene not part of the ABA signaling network | 407 | 18522 | 594 |
| Gene part of the ABA signaling network | 13 | 103 | 21 |

**Table S7.** Pearson's chi-square (Pearson) and the G (Likelihood ratio) for the independence of the known ABA signaling genes and those differentially expressed under 3 hours of ABA treatment in A. thaliana.

| Test of association | Statistic | Degrees of freedom | P value | Effect size measure | Effect size |
| --- | --- | --- | --- | --- | --- |
| G-test | 58.64 | 2 | 1.8463e-13 | Contingency coefficient | 0.07 |
| Pearson's $\chi^{2}$ test | 106.13 | 2 | < 2.22e-16 | Cramer's V | 0.07 |

**Table S8.** Regulatory interactions with known ABA transcription factors among differentially expressed genes. Known? refers to has whether the target is known member of the ABA signaling pathway.

| TF | Target | *B. napus* TF | *B. napus* targets | Type | Known? |
| --- | --- | --- | --- | --- | --- |
| AtMYC2 | ADH1 | BnaC08g07580D, BnaA05g18020D | BnaC06g37860D, BnaA07g33310D | Activation | No |
| AtMYC2 | RD22 | BnaC08g07580D, BnaA05g18020D | BnaC07g29150D, BnaA06g39340D | Activation | No |
| AtMYC2 | CAB1 | BnaC08g07580D, BnaA05g18020D | BnaC03g59520D | Repression | No |
| HY5 | At1g76180 | BnaA10g21200D | BnaC06g36880D, BnaA07g21490D, BnaA07g32420D, BnaA02g36030D, BnaC06g21970D | Unknown | No |
| HY5 | At2g37180 | BnaA10g21200D | BnaC04g08090D, BnaA05g07290D, BnaC04g08100D, BnaA05g07300D | Unknown | No |
| HY5 | At4g33210 | BnaA10g21200D | BnaA01g03630D, BnaC01g04970D | Unknown | No |
| HY5 | At1g19000 | BnaA10g21200D | BnaC08g18480D, BnaA08g22290D, BnaC08g36920D, BnaA09g44370D | Unknown | No |
| HY5 | RD26 | BnaA10g21200D | BnaA01g16400D, BnaC07g40860D, BnaA03g48570D | Unknown | Yes |
| HY5 | At3g16320 | BnaA10g21200D | BnaC05g37040D | Unknown | No |
| HY5 | CHI | BnaA10g21200D | BnaA09g34840D, BnaC08g26010D | Activation | No |
| HY5 | ELF4 | BnaA10g21200D | BnaA05g05560D | Activation | No |

**Table S9.** Binding site sequences over-represented in the putative promoter regions of genes up-regulated by ABA, along with the corresponding transcription factors.

| Binding site sequence (BSS) | Number of promoter regions of up-regulated genes with BSS (out of 7669) | Total number of promoter regions with BSS (out of 101040) | Adjusted pval | *A. thaliana* transcription factor(s) that bind to the BSS | Orthologous *B. napus* transcription factors | Is the transcription factor part of the ABA signaling network? |
| --- | --- | --- | --- | --- | --- | --- |
| ABRE binding site motif | 2625 | 19775 | 2.897e-216 | ABF4 (AT3G19290) | BnaCnng41320D, BnaA01g26200D, BnaC01g43800D, BnaA03g35190D, BnaA05g20870D, BnaC05g33570D | Yes |
| ABFs binding site motif | 2003 | 13791 | 6.162e-200 | ABF1 (AT1G49720), ABF2 (AT1G45249) | BnaC06g02640D, BnaA06g03040D, BnaC06g00420D, BnaA10g28780D | Yes, Yes |
| ABRE-like binding site motif | 6442 | 71043 | 4.318e-181 | NA (NOTAVAILABLE) | NA | No |
| CBF2 binding site motif | 960 | 6780 | 8.816e-81 | ATCBF2 (AT4G25470) | BnaA08g30910D | No |
| GBF1/2/3 BS in ADH1 | 960 | 6780 | 8.816e-81 | AtGBF1 (AT4G36730), ATBZIP54 (AT4G01120), GBF3 (AT2G46270) | BnaC03g61840D, BnaC01g02130D, BnaA01g01100D, BnaA08g15400D, BnaCnng01910D, BnaA09g00170D, BnaC04g01070D, BnaC03g25660D, BnaA05g01520D | No, No, No |
| ERF1 BS in AtCHI-B | 5106 | 57711 | 5.664e-68 | ATERF1 (AT3G23240) | BnaA07g06760D, BnaC07g08360D, BnaA01g23940D | No |
| TGA1 binding site motif | 1633 | 15985 | 4.986e-38 | TGA1 (AT5G65210) | BnaC09g06840D, BnaAnng04720D, BnaC02g43620D, BnaA09g07120D, BnaA06g24140D, BnaC03g49070D | No |
| RAV1-B binding site motif | 6168 | 77449 | 3.646e-15 | AtRAV2 (AT1G68840) | BnaC02g18650D, BnaA02g14040D | No |
| HSEs binding site motif | 2281 | 28000 | 0.001116 | AT-HSFC1 (AT3G24520) | BnaC07g07130D, BnaA03g37460D, BnaC03g43990D, BnaA07g05580D | No |
| DREB1&2 BS in rd29a | 204 | 2036 | 0.001853 | ATCBF2 (AT4G25470) | BnaA08g30910D | No |
| AtMYB2 BS in RD22 | 4689 | 59738 | 0.004914 | ATMYB2 (AT2G47190) | BnaA05g00710D, BnaC04g51450D | Yes |
| E2F-varient binding site motif | 211 | 2182 | 0.01041 | NA (AT2G36011) | NA | No |
| VOZ binding site | 155 | 1541 | 0.01253 | ATVOZ1 (AT1G28520) | BnaC05g21930D, BnaC03g58740D, BnaA08g18270D, BnaA09g27210D | No |
| ARF binding site motif | 7433 | 97264 | 0.03437 | ARF1 (AT1G59750) | BnaC01g28340D, BnaA01g35830D | No |

**Table S10.** Binding site sequences over-represented in the putative promoter regions of genes down-regulated by ABA, along with the corresponding transcription factors.

| Binding site sequence (BSS) | Number of promoter regions of up-regulated genes with BSS (out of 7669) | Total number of promoter regions with BSS (out of 101040) | Adjusted pval | *A. thaliana* transcription factor(s) that bind to the BSS | Orthologous *B. napus* transcription factors | Is the transcription factor part of the ABA signaling network? |
| --- | --- | --- | --- | --- | --- | --- |
| ERF1 BS in AtCHI-B | 2766 | 57711 | 5.794e-06 | *ATERF1 (AT3G23240)* | BnaA07g06760D, BnaC07g08360D, BnaA01g23940D | No |
